# Homologous tropomyosins from vertebrate and invertebrate: Recombinant calibrator proteins in functional biological assays for tropomyosin allergenicity assessment of novel animal foods

**DOI:** 10.1111/cea.13503

**Published:** 2019-10-11

**Authors:** Julia Klueber, Joana Costa, Stefanie Randow, Françoise Codreanu‐Morel, Kitty Verhoeckx, Carsten Bindslev‐Jensen, Markus Ollert, Karin Hoffmann‐Sommergruber, Martine Morisset, Thomas Holzhauser, Annette Kuehn

**Affiliations:** ^1^ Department of Infection and Immunity Luxembourg Institute of Health Esch‐sur‐Alzette Luxembourg; ^2^ Department of Dermatology and Allergy Center Odense Research Center for Anaphylaxis University of Southern Denmark Odense C Denmark; ^3^ REQUIMTE‐LAQV/Faculdade de Farmácia da Universidade do Porto Porto Portugal; ^4^ Division of Allergology Paul‐Ehrlich‐Institut Langen Germany; ^5^ National Unit of Immunology and Allergology Centre Hospitalier de Luxembourg Luxembourg Luxembourg; ^6^ RAPID TNO Zeist The Netherlands; ^7^ Department of Pathophysiology and Allergy Research Medical University of Vienna Vienna Austria; ^8^ Present address: Unité d’Allergologie CHU Angers Angers France

**Keywords:** allergenicity assessment, basophil activation test, chicken, rat basophil leukaemia cell mediator release, shrimp, shrimp allergy, tropomyosin

## Abstract

**Background:**

Novel foods may provide new protein sources for a growing world population but entail risks of unexpected food‐allergic reactions. No guidance on allergenicity assessment of novel foods exists, while for genetically modified (GM) crops it includes comparison of sequence identity with known allergens, digestibility tests and IgE serum screening.

**Objective:**

As a proof of concept, to evaluate non‐/allergenic tropomyosins (TMs) regarding their potential as new calibrator proteins in functional biological in vitro assays for the semi‐quantitative allergy risk assessment of novel TM‐containing animal foods with mealworm TM as an example.

**Methods:**

Purified TMs (shrimp, *Penaeus monodon*; chicken *Gallus gallus*;* E coli* overexpression) were compared by protein sequencing, circular dichroism analysis and in vitro digestion. IgE binding was quantified using shrimp‐allergic patients' sera (ELISA). Biological activities were investigated (skin testing; titrated basophil activation tests, BAT), compared to titrated biological mediator release using humanized rat basophil leukaemia (RBL) cells.

**Results:**

Shrimp and chicken TMs showed high sequence homology, both alpha‐helical structures and thermal stability. Shrimp TM was stable during in vitro gastric digestion, chicken TM degraded quickly. Both TMs bound specific IgE from shrimp‐allergic patients (significantly higher for shrimp TM), whereas skin reactivity was mostly positive with only shrimp TM. BAT and RBL cell assays were positive with shrimp and chicken TM, although at up to 100‐ to 1000‐times lower allergen concentrations for shrimp than chicken TM. In RBL cell assays using both TM as calibrators, an activation of effector cells by mealworm TM similar to that by shrimp TM confirmed the already reported high allergenic potency of mealworm TM as a novel protein source.

**Conclusions & clinical relevance:**

According to current GM crops' allergenicity assessment, non‐allergenic chicken TM could falsely be considered an allergen on a weight‐of‐evidence approach. However, calibrating allergenic potency in functional BAT and RBL cell assays with clinically validated TMs allowed for semi‐quantitative discrimination of novel food protein's allergenicity. With TM calibration as a proof of concept, similar systems of homologous protein might be developed to scale on an axis of allergenicity.

## 1 INTRODUCTION

The United Nations forecast a growth of the world population by 50% in the period from 2000 to 2050 which is expected to lead to a pressing need for more food production but also the introduction of novel types of foods in order to address a shortage of protein sources.^1^, ^2^ Novel foods, defined differently in different countries but in the European Union as any kind of food not yet been used in human diet to a significant degree before 1997, are entering continuously the market.^3^ Prominent examples of novel foods from animal origin are edible insects or exotic meats (eg crocodile or kangaroo meat) and from plant origin, algae‐related food (eg spirulina) or novel seeds (eg chia seeds).^4^, ^5^, ^6^, ^7^ In the absence of studies on epidemiology and natural history of allergic reactions to novel foods, these foods bear possible risk for food‐allergic reactions in allergic patients due to clinical cross‐reactions as well as in previously tolerant individuals due to de novo sensitization.^8^, ^9^ However, there is no generally accepted strategy for the allergenicity assessment of novel foods prior to market launch.^10^

Food allergy emerged over the past thirty years as an important public health problem, in a second wave of the “allergy epidemic” succeeding the first respiratory epidemic.^11^ About 20 million people in Europe suffer from food allergies, defined as chronic immune‐mediated adverse reactions to otherwise harmless food proteins.^12^, ^13^ IgE‐mediated reactions are the most common and best‐understood food allergy type, manifesting as a multi‐organ disease and varying from mild clinical symptoms to severe anaphylaxis.^14^, ^15^ Today, effective management of food‐allergic patients relies on strict avoidance diet of the culprit food source of origin including other cross‐reactive foods.^16^ Successful food allergen avoidance is a challenging task involving not only the patient but also the patient's personal/professional environment as well as other stakeholders, public authorities and food industry.^17^ Achieving food allergen avoidance becomes even more complex when novel foods suddenly become available on the consumer market.^8^

Dietary proteins are the main drivers of food‐allergic reactions, being responsible for both allergic sensitization (eg via ingestion, inhalation and cutaneous uptake) as well as clinical manifestations in previously sensitized patients.^14^, ^18^ Potent food allergens are often highly stable proteins and beyond, impaired gastric digestion and increased intestinal permeability have been associated with clinical food allergy, suggesting particular bioavailability of food antigens in allergic conditions.^19^, ^20^ Homologous panallergens from closely related sources are often cross‐reactive, such as crustacean tropomyosins and fish parvalbumins.^21^, ^22^, ^23^ However, homologous proteins might also differ by their allergenic potency, especially when they originate from distantly related sources.^24^

Tropomyosins (TMs) are known panallergens in arthropods and major food allergens in, for example various species of shellfish, molluscs and the fish parasite Anisakis as well as respiratory minor allergens from environmental origin (eg mites and cockroaches).^23^ Fifteen arthropod TMs have been registered as food allergens according to the criteria set by the WHO/IUIS allergen nomenclature sub‐committee (http://www.allergen.org, retrieved 01 March 2019). They are highly homologous alpha‐helical proteins sharing characteristic rod‐shaped structures of two parallel helices. Allergenic TMs are strong food allergens and highly cross‐reactive within the invertebrate group. IgE reactivity to TM is associated with severe symptoms in shrimp‐allergic patients.^25^ Recently, TM from yellow mealworm, the larvae of the beetle *Tenebrio molitor*, was identified as a main allergen from this novel food source.^4^, ^26^ Although TMs are highly conserved proteins throughout the animal kingdom, only homologues from invertebrate source are verified allergens while vertebrate TMs, such as mammalian and avian TMs including chicken TM, are generally considered as non‐allergenic.^27^ Structural relationship of proteins or a certain degree of amino acid (aa) identity increases the probability of allergic cross‐reactivity. However, mere aa identity and structural homology, essentially when low thresholds of criteria are applied (eg 35% sequence identity to a known allergen using a sliding window of 80 aa), are weak predictors in allergen risk assessment of novel foods, and additional tests are needed to assess their allergenic potential.^10^

For genetically modified (GM) food crops, guidance based on a decision tree for the allergen risk assessment exists and includes, for example analysis whether the source of the protein is known to be allergenic or not, comparison of sequence identity with known allergens, digestibility tests and IgE serum screening (eg with sera of patients allergic to the source of the protein).^28^, ^29^ By contrast, there is no generally accepted strategy of how to perform allergenicity assessment of novel foods.^10^ Recently, an European food allergy expert consortium (COST action FA1402, ImPARAS, 2014‐2018) identified important gaps in the safety assessment of novel foods with major weaknesses in guidance on allergenicity and immunogenicity assessment of food sources.^8^, ^9^, ^10^ A multidisciplinary strategy has been identified for future systematic and comprehensive food risk assessment, with emphasis on using well‐characterized pairs of homologous proteins as opponents on an allergenicity scale, in order to validate applied methodologies and to calibrate the assays for those cases where the putative novel food allergen is known to have allergenic/non‐allergenic homologues.^10^ In the present study, we followed the available guidance on the allergenicity assessment of GM crops, extended it for in vitro biological potency assays and used TMs from an invertebrate origin, the well‐known food allergen from black tiger shrimp (*Penaeus monodon*) muscle, and TM from a vertebrate origin, a non‐allergenic molecule expressed in chicken (*Gallus domesticus*) muscle, as a pair of homologous proteins. First, we characterized the two TMs at the biomolecular level and second used them in differential in vitro assays to assess their relative allergenic potency in shrimp‐allergic individuals. The overall goal was to evaluate whether the pair of homologous TMs with divergent allergenic potential is suitable to calibrate in vitro biological potency assays in order to assess semi‐quantitatively and confirm the allergenic potency of a known novel animal food protein, namely tropomyosin of yellow mealworm. Results are evaluated and discussed in the light of existing allergenicity assessment guidance.

## 2 METHODS

### 2.1 Shrimp‐allergic patients and sera

Twenty shrimp‐allergic patients (12 male, 8 female; mean age 18.8 years) were recruited at the National Unit of Immunology and Allergology, Central Hospital of Luxembourg (CHL). Criteria for selection were a convincing clinical history of shrimp allergy and positive prick‐to‐prick tests (PTP) with shrimp as well as specific IgE higher than 0.1 kU_A_/L for shrimp extract and tropomyosin Pen a 1 (ImmunoCAP, Phadia‐Thermofisher). All patients developed allergic symptoms upon exposure to shrimp (ingestion and inhalation/skin contact) on several occasions. Shrimp‐allergic patients tolerated chicken meat (regular consumption, ≥1×/month). PTP were performed with cooked shrimp muscle and in single cases (randomly selected: patient no. 1, 2, 6, 9), with cooked chicken muscle. Histamine was used as positive control. A positive PTP response was scored when the weal diameter was 3 mm larger than the negative control (glycerine‐containing saline). Patients without severe history of shrimp allergy and positive Pen a 1‐titres (1‐50 kU_A_/L) were asked for further skin testings. Single patients (n = 6) agreed to be tested for skin reactivity with increasing concentrations of native, purified TM (0.1, 1, 10, 50 μg allergen/mL) diluted in sterile saline containing 0.03% human serum albumin (ALK, Denmark).^24^ Skin testing was stopped at the protein concentration where a positive reaction was scored. All patients were also examined as to house dust mite (HDM) allergy (medical history, specific IgE for HDM extract and Der p 10). Two individuals with allergies other than to shrimp were used as negative controls in IgE testing (IgE ELISA, effector cell reactivity tests) (Table S1). The study was approved by the National Committee for Medical Research Ethics in Luxembourg (Ref. CNER approval No. 201307/04) and informed consent was given by all the patients.

### 2.2 Generation and purification of natural and recombinant tropomyosins (TM)

Black tiger prawn (*Penaeus monodon*) and chicken (*Gallus gallus*; breast, leg) TM were isolated according to published procedures,^30^ modified as detailed below. Proteins were extracted in chromatography buffer 20 mmol/L Tris, pH 8 and heated (80°C, 5 minutes). Supernatants were separated by anion exchange chromatography (Resource Q, GE Healthcare). Proteins were eluted in a salt gradient (20 mmol/L Tris, 1 M NaCl, pH 8). The buffer of TM‐containing fractions was exchanged to 20 mmol/L 2‐(N‐Morpholino) ethanesulphonic acid (MES), pH 5.8. Fractions were further purified by cation exchange (Resource S, GE Healthcare; gradient using 20 mmol/L MES, 1 M NaCl, pH 5.8).

Shrimp TM Pen m 1 (A1KYZ2) was expressed in *E coli* M15 and chicken TM α‐1 chain isoform X1 (P04268) in *E coli* BL21 (DE3).^31^, ^32^ Briefly, recombinant protein expression was induced using isopropyl β‐D‐1‐thiogalactopyranoside (IPTG) and affinity‐tagged TMs purified by immobilized metal ion affinity chromatography. Purified proteins were stored in phosphate‐buffered saline (PBS) buffer, pH 7.2 until use (aliquots, −20°C). Native mealworm TM was prepared during a previous study.^33^

### 2.3 Protein sequence determination by Edman sequencing and mass spectrometry analysis

N‐terminal sequences of TMs were analysed by automated Edman degradation (Procise 49X HT protein sequencer, Applied Biosystems). Matrix‐assisted laser desorption/ionization time‐of‐flight mass spectrometer (MALDI TOF; Bruker) analysis identified tryptic digested proteins by peptide mass fingerprints (PMF) and comparison with the MASCOT 2.0 search engine (Matrix Science) in the NCBInr database.^34^

### 2.4 Secondary protein structure determination by circular dichroism

Tropomyosins were measured in 20 mmol/L KH_2_PO_4_ pH 7.2 to establish their circular dichroism (CD) spectra using the Chirascan CD spectrometer (Applied Photophysics). Thermal sensitivity was assessed by ramping temperatures (20°C to 95°C to 20°C). Far‐ultraviolet CD spectra were recorded at 180‐260 nm (1 nm bandwidth, 0.5 seconds interval, 5 repeats). The read‐out was converted with respective protein details into degrees*cm^2^*dmol^–1^. GLOBAL 3 and DICHROWEB software were used to analyse and interpret CD spectra, measured as a function of temperature.

### 2.5 In vitro digests

Simulated digestion was performed as reported previously,^35^ with further modifications as published for successive gastric and intestinal digests according to the international consensus paper published by Minekus et al,^36^ in terms of adjusted pH and applied incubation times. Briefly, protein extracts were incubated with pepsin (Sigma‐Aldrich) at a final ratio of 1 U enzyme/µg protein extract in simulated gastric fluid, pH 3 for 2 hours. The last sample from the gastric digest was mixed 1:1 with pancreatin (based on trypsin activity at 100 U/mL) and 10 mmol/L bile salts (both Sigma‐Aldrich) for two hours in simulated intestinal fluid, pH 7. Samples were drawn at T0, T1, T2, T5, T10, T20, T30, T60, T90 and T120 min during each digestion phase, following by analysis on SDS‐PAGE and immunoblot.

### 2.6 Immunoblot and ELISA analyses using commercial antibodies and patient sera

Tropomyosins samples were separated by SDS‐PAGE/Coomassie/silver stain, in order to revise protein purity and protein size.^37^ Protein identity was confirmed by immunodetection in immunoblot and enzyme‐linked immunosorbent assay (ELISA). Polyclonal rabbit IgG‐antibodies were used to detect shrimp (PA‐SHM; Indoor Biotech) or chicken TM (ab11190; Abcam), each antibody diluted 1:10 000 in immunoblot and 1:5000 in ELISA analysis, followed by secondary antibody incubation (1:10 000; anti‐rabbit IgG‐antibody labelled with alkaline phosphatase; Sigma‐Aldrich).

IgE‐immunoblot analysis of shrimp total protein extract was done using 20 shrimp‐allergic patients.^31^ 20 µg/cm shrimp total protein/gel slot was separated in SDS‐PAGE and blotted onto a nitrocellulose membrane. After blocking with 3% bovine serum albumin (BSA) and 0.3% Tween 20 in Tris‐buffered saline (50 mmol/L Tris, pH 7.4), 50 µL of sera were diluted ad 600 µL TBS and added to individual 2 mm wide membrane strips. Bound IgE was detected using 1:1000 diluted (TBS) monoclonal mouse anti‐human IgE antibody (Southern Biotech), conjugated with alkaline phosphatase, and using 5‐bromo‐4‐chloro‐3‐indolyl phosphate/nitro blue tetrazolium (Sigma) as precipitating substrate.

IgE ELISA was used to quantify IgE titres in patient sera.^34^ In short, patient sera were fivefold, 10‐ and 20‐fold diluted in blocking buffer containing 3% bovine serum albumin (BSA). Five sera of non‐atopic individuals were used as negative controls, resulting in a 10‐fold lower mean background than the cut‐off value (0.1 kU_A_/L).

### 2.7 Basophil activation test

Basophil activation was assessed using the Flow‐CAST kit^®^ (BÜHLMANN Laboratories AG, Swiss).^24^ We included seven representative patients, patients no. 1‐7, Table 1, with various clinical profiles (Pen a 1‐specific IgE titres low to high; clinical symptoms mild to severe). Briefly, basophils from fresh blood were stimulated with serial protein concentrations (0.1‐10 000 ng/mL) and following, the percentage of activated (CD63^+^ CCR3^+^) basophils upon stimulation measured by flow cytometry (cut‐off for positivity ≥15% of CD63^+^ basophils compared to total basophils). Data acquisition/analysis was performed using BD FACSDiva software (BD Biosciences) and Kaluza (Beckman Coulter) software, respectively.

**Table 1:** Demographic and clinical data from shrimp‐allergic patients

No.	Gender/Age	Symptoms[Link]	Prick‐to‐prick test[Link] (mm)	IgE titres[Link] (kU_A_/L)		
				Total	Shrimp	Pen a 1
1	M/17	A, AE	16	110	62	34
2	M/9	V	20	1455	11	6.5
3	M/17	AE, U	14	265	5	3
4	M/16	AP, U	11	4640	12	10
5	M/6	AE, OAS, U	12	240	100	100
6	M/10	OAS, U	3	1432	5.4	1.7
7	F/13	AE	8	447	5.3	4.1
8	F/40	A, AE, OAS	7	62	1.9	1.6
9	F/15	AE, Ai, OAS	16	1055	42	31
10	M/13	U	18	95	0.5	0.4
11	F/21	AE, OAS	6	361	100	100
12	M/7	OAS	5	280	3.1	2.1
13	F/20	OAS	11	1235	37	10
14	M/19	AE, OAS	6	6780	100	100
15	M/14	U	15	320	0.3	0.4
16	F/52	A, AE, U	7	1220	1.4	0.2
17	M/41	U	8	225	0.4	0.5
18	M/15	AP	11	402	2.5	2
19	F/16	U	20	749	26	40
20	F/52	A, AE, U	6	111	3.1	1.4
Mean	20.7	‐	11	1074	25.9	22.4
Median	16	‐	11	382	5.4	3.6

Symptoms: A, asthma (ingestion); Ai, asthma (inhalation); AE, angioedema; AP, abdominal pain; OAS, oral allergy syndrome; U, urticaria; V, vomiting.

Prick‐to‐prick with cooked shrimp muscle, weal diameter in millimetre.

IgE determination by ImmunoCAP: shrimp, shrimp extract (f24); Pen a 1, shrimp tropomyosin (f351).

### 2.8 Mediator release assay

Mediator release assays were performed using the rat basophilic leukaemia (RBL)–2H3 cell line transfected with the α‐chain of the human high‐affinity IgE receptor.^38^ Briefly, patient sera (patients no. 1‐20, Table 1; 50 μL/well; dilution 1/11) were incubated with the cells (clone RBL‐703/21). Serial dilution of all proteins (0.001‐10 000 ng/mL) was used to induce antigen‐specific release quantified by measuring the enzymatic activity of β‐hexosaminidase. Releases were expressed as the percentage of release from cells sensitized with serum in relation to the total release obtained by lysis of all cells (cut‐off for positivity ≥10% of positive release compared to total release). Adjustment for spontaneous release (serum added without allergen) was carried out.

### 2.9 Statistical analyses

IgE results were plotted using GraphPad Prism 5 (GraphPad software). Differences in IgE titres were assessed using the nonparametric Kruskal‐Wallis test and the Mann‐Whitney *U* test.

## 3 RESULTS

### 3.1 Shrimp‐allergic patients tolerate chicken meat

All patients experienced multiple allergic episodes upon shrimp ingestion with clinical symptoms detailed in Table 1. Skin prick tests with cooked shrimp muscle were positive in all individuals (mean weal diameter: 11 mm). Serum IgE antibodies were positive for shrimp extract as well as shrimp TM Pen a 1 (mean titre 25.9 and 22.4 kU_A_/L, respectively). All participants tolerated chicken meat (regular consumption, min. 1×/month). Skin test with cooked chicken meat was negative, as assessed in randomly selected patients (patient no. 1, 2, 6, 9). Most patients (17/20; 85%) had also an allergy to house dust mites (HDM) with characteristic symptoms (eyes, nose and in some cases, respiratory tract) as well as positive serum IgE titres (mean titre HDM extract 47.1 kU_A_/L, HDM TM Der p 10 39.9 kU_A_/L, respectively) (Table S2).

### 3.2 In‐/vertebrate TM: similar thermal but divergent in vitro digestion stability

**Figure 1:**
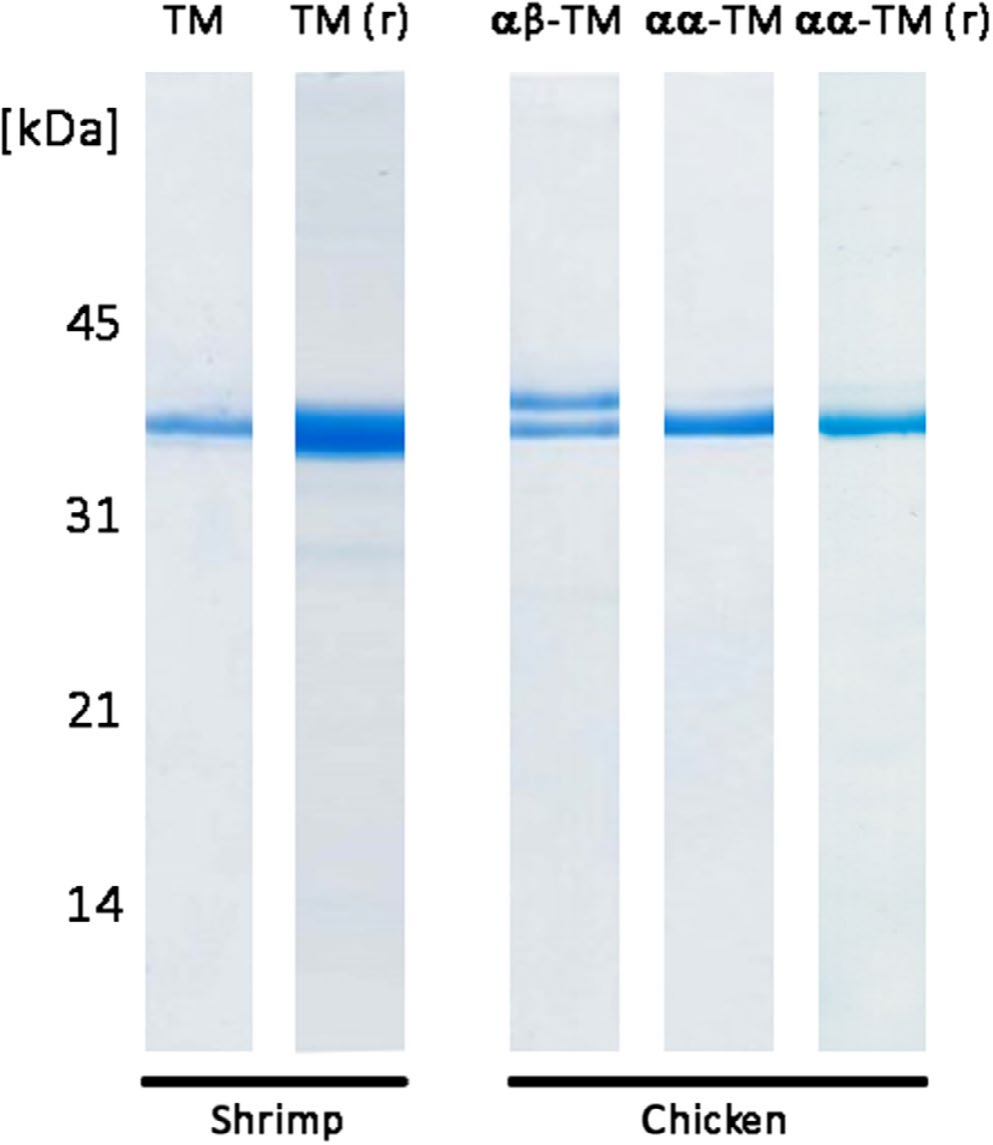
Shrimp and chicken tropomyosins, both native and recombinant, were purified to homogeneity. Native shrimp tropomyosin was found to be a homodimer (single band) while chicken tropomyosin from breast muscle was mostly a α/α‐homodimer and chicken tropomyosin from leg muscle a α/β‐heterodimer (double band). r, recombinant protein

Tropomyosins from shrimp and chicken were detected in extracts prepared from muscle using specific anti‐TM antibodies (Figure S1). Both homologues proved to be heat‐stable, being still detectable in samples after extract heating (80°C, 10 minutes). Shrimp and chicken TMs were purified to homogeneity by column chromatography. Native shrimp TM migrated as a single band in SDS‐PAGE (Figure 1). Chicken TM purified from breast migrated mostly as a single protein band while TM purified from chicken leg appeared as a clear double band. N‐terminal sequencing of shrimp TM‐ as well as the higher chicken TM‐band revealed no clear sequence. The N‐terminus of the lower chicken TM (identified peptide: LDKENALDRAEQAEAD) confirmed the identity of the protein (16/16 aa, 100% identity to chicken TM α XP_015134264). Mass spectrometric (MS) analysis identified the purified shrimp protein as allergen Pen m 1 (95.1% sequence coverage to ADM34184.1), the upper chicken band as TM β‐chain (73.2% sequence coverage to P19352.1) and the lower chicken band as TM α‐chain (79.2% sequence coverage to P04268.2) (Tables S3‐S5). Accordingly, TM purified from chicken leg was concluded to be a TM α/β‐heterodimer and TM purified from chicken breast to be mostly an α/α‐homodimer. The overall protein identity between shrimp Pen m 1 and the chicken homologues ranged between 55% and 59% for the chicken TM β‐chain and the chicken TM α‐chain, respectively (NCBI‐BLASTp sequence comparison) (Figure S2). Both chicken TM sequences had up to 70% best sequence identities within a 80 mer sliding window for TM allergens Pen m 1, Lit v 1 and Cra c 1 (http://www.allergenonline.org). Recombinant shrimp Pen m 1 and chicken TM α‐isoform were purified after overexpression in *E coli* (Figure 1). Antibody‐based detection as well as N‐terminal sequencing and MS analysis, followed by sequence comparison to the respective database entries (data not shown), further confirmed the peptide sequence of the purified recombinant proteins as a quality control.

Secondary protein structures were compared by circular dichroism (CD) analysis. Alpha‐helical structures were found for both shrimp and chicken homologues (native, recombinant), reflected by CD curves with two characteristic minima at 208 and 222 nm (Figure S3). All proteins unfolded upon heating at 95°C and after chilling, refolded to alpha‐helical structures—confirming the thermal stability of TMs from both shrimp and chicken origin.

The stability of shrimp and chicken TMs towards in vitro digestion was analysed in sequential protein extract digests mimicking a gastric followed by an intestinal digestion phase. Under the applied conditions, Pen m 1 was detectable by immunoblot during the gastric phase (last signal at 90 minutes), first at the expected molecular weight and following, at lower molecular weight corresponding to a 30 kDa‐fragment of the natural allergen (Figure S4). The specific anti‐TM antibody recognized chicken TM only until 5 minutes of gastric digest, revealing a low stability towards pepsin proteolysis in comparison with the shrimp homologue. All samples from intestinal digest of both shrimp and chicken extract were negative in immunoblots with anti‐TM antibodies (data not shown).

### 3.3 Low IgE titres to chicken proteins and purified chicken TM

**Figure 2:**
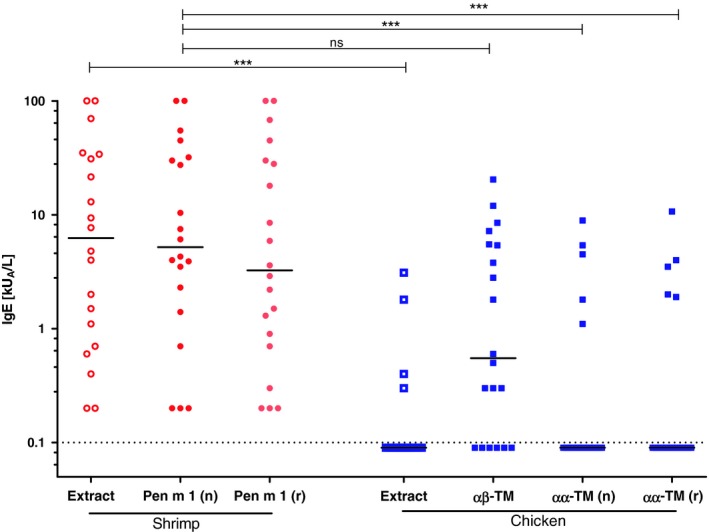
IgE levels to chicken tropomyosins are significantly lower than IgE levels to shrimp tropomyosins. IgE signal intensity is reflected in a grading log10 scale for concentrations of measured specific IgE (kU_A_/L), medians are shown. ^***^ *P* < .001; dotted line, cut‐off for positivity; n, native protein; ns, not significant; Pen m 1, shrimp tropomyosin; r, recombinant protein; TM, tropomyosin

Levels of specific IgE to shrimp extract, chicken extract, mealworm extract, natural and recombinant shrimp and chicken TM were compared using IgE ELISA analysis (Table S6). Specific IgE to shrimp extract was found in all study participants by IgE ELISA (0.2‐100 kUA/L median 6.3 kUA/L) (Table S6). All patients were also positive in the IgE ELISA test with shrimp TM, both native and recombinant proteins (0.2‐100 kUA/L, median 5.2 kUA/L and 0.2‐100 kUA/L, median 3.3 kUA/L, respectively) (Figure 2). Only 20% (4/20) of the patients had IgE to chicken extract (0.3‐3.1 kUA/L, median 0.4 kUA/L) while 70% had specific IgE to chicken leg TM, the heterodimeric αβ‐molecule (0.3‐20.4 kUA/L, median 3.5 kUA/L). TM purified from chicken breast (αα‐homodimers), both native and recombinant molecules, was recognized by IgE antibodies of 25% of the study cohort (1.1‐8.9 kUA/L, median 1.2 kUA/L and 1.9‐10.7 kUA/L, median 1.2 kUA/L, respectively). Median titres of specific IgE to shrimp extract and (r)Pen m 1 were significantly higher compared to chicken extract and the TM homodimer.

As several patients had low IgE to purified shrimp TM, IgE immunoblots were performed with shrimp extract in order to revise the role of other allergens. For most patients (17/20; 85%), IgE immunoblots with shrimp extract revealed weak to strong signals of antibody binding to ca. 38 kDa‐shrimp TM (Figure S5), thus confirming the relevance of this major allergen for the present patient cohort. Very weak anti‐TM‐sIgE signals were detected for patient no. 16 and 17. Four patients (20%; no. 1, 2, 5, 13) had additional IgE reactivity to 20 kDa‐allergens that were identified by MALDI‐MS analysis as sarcoplasmic calcium‐binding protein (data not shown), another well‐established shrimp allergen.^25^

### 3.4 Low skin prick reactivity to chicken TM in shrimp‐allergic patients

**Figure 3:**
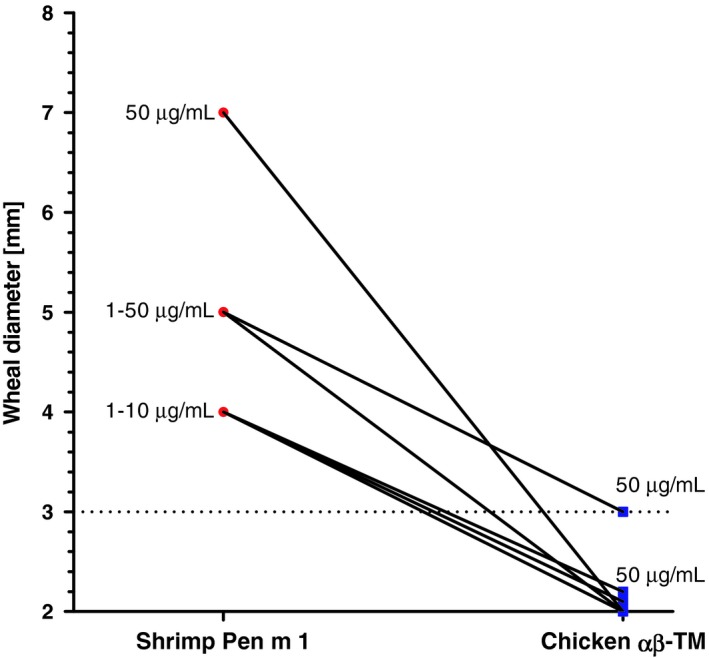
Skin prick tests are positive in all tested shrimp‐allergic patients using purified shrimp tropomyosin (1‐50 µg/mL). Only one patient had a weak skin reaction at the highest concentration of chicken tropomyosin. Pen m 1, shrimp tropomyosin; TM, tropomyosin

Purified native TM from shrimp induced positive skin reactions in all of the tested shrimp‐allergic patients (weal diameter: mean 4.8 mm) (Figure 3). Only one patient had a weak positive skin test with chicken leg TM, at the highest concentration tested (weal diameter 3 mm). Detailed results of the SPT are shown in the online Table S7. The negative control (saline) did not induce any skin reaction.

### 3.5 Chicken TM has low capacity to activate basophils in vitro

**Figure 4:**
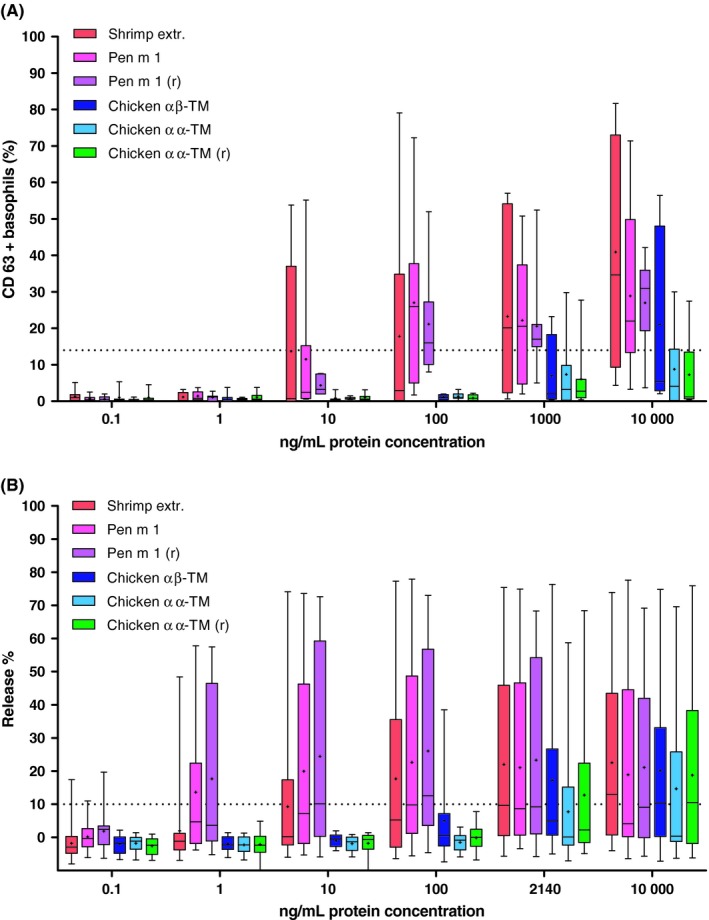
A, Basophil activation is induced efficiently at low protein concentrations (≤100 ng/mL) by shrimp tropomyosin but not chicken homologues (n = 7 shrimp‐allergic patients). B, Shrimp tropomyosin has a high capacity to induce at low concentrations (≤10 ng/mL) histamine release from RBL cells previously sensitized with sera from 20 shrimp‐allergic patients, as compared to chicken proteins

Tropomyosins from chicken (native, recombinant) showed a drastically lower potency to activate effector cells compared with shrimp homologues (Figure 4A,B). Upon stimulation with 100 ng/mL of TM, the median percentage of patients’ CD63^+^ basophils was 16%‐26% with shrimp proteins compared to <2% with chicken homologues (Figure 4A). 10 000 ng/mL chicken TM resulted in an activation that hardly reached the minimum positivity lower threshold of 15% basophil activation. In comparison, 100‐fold less shrimp tropomyosin easily exceeded this level. Thus, the capacity to activate basophils using shrimp versus chicken TM differed largely. In basophil activation tests using patients cells, all patients responded positively to the stimulation with an anti‐FcɛRI monoclonal antibody (35.6%‐97.1%, median 62.5%), while 4/7 patients responded to fMLP stimulation (9.3%‐77.0%, median 23.6%). All control assays with shrimp‐tolerant individuals revealed no basophil activation in response to stimulation with TM in a concentration of up to 10 µg/mL (data not shown).

In comparison with shrimp proteins, chicken TM also revealed low potency to induce β‐hexosaminidase release from humanized rat basophils that were passively sensitized with IgE from patient sera. Following stimulation with 100 ng/mL of protein, the median percentage of histamine release was 10%‐13% with shrimp TM (range of histamine release: Pen m 1, 0.7%‐77.9%; rPen m 1, 0.2%‐73.0%) compared to <0.8% with chicken homologues (Figure 4B). A comparable median mediator release of 10% was obtained after increasing the chicken TM concentration to 10 000 ng/mL in comparison with 100 ng/mL for shrimp TM. Differences in statistical significance, high capacity to induce mediator release using shrimp but not chicken TM, were found for the protein concentrations tested (eg Pen m 1 (r) vs chicken α/α *P* = .0087 Pen m 1 (r) vs chicken α/β *P* = .0260; n = 20). No mediator release was induced when using two sera from tolerant controls (Table S1) for rat basophil sensitization (data not shown).

### 3.6 Application of shrimp and chicken TM in allergenicity assessment assay

**Figure 5:**
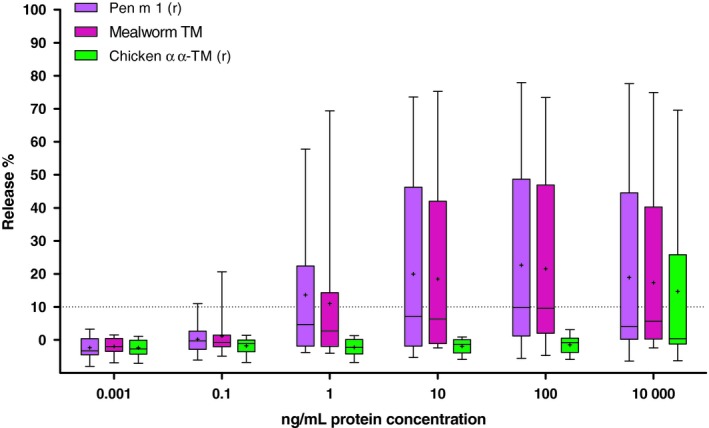
The allergenic shrimp tropomyosin and the non‐allergic chicken homologue (both recombinant) are opponents on an allergenicity scale using the histamine release assay with sensitized RBL cells and allow to assess the allergenic potency of mealworm tropomyosin as a novel food allergen

Recombinant TM from shrimp (Pen m 1) and chicken (α/α homodimer) was used to scale the relative allergenic potency of mealworm TM, a novel food allergen, using β‐hexosaminidase release from rat basophilic leukaemia cells expressing the human high‐affinity IgE receptor (Figure 5). Upon stimulation with 100 ng/mL of TM, the median percentage of 10%‐11% β‐hexosaminidase release was significantly higher for shrimp and mealworm TM, as compared to <0.2% with chicken TM (*P* = .0411). Indeed, this allowed confirming the high allergenic potency of mealworm TM, similar to shrimp TM, by using shrimp and chicken homologues as calibrator proteins in this cellular in vitro assay.

## 4 DISCUSSION

In this study, we evaluated two homologous proteins, a pair of tropomyosins (TM) consisting of the highly allergenic shrimp Pen m 1 and the non‐allergenic counterpart from chicken muscle. We investigated their potential use as calibrator molecules for the assessment of protein allergenicity in in vivo skin prick testing, in vitro IgE‐binding capacity, ex vivo basophil activation and in vitro mediator release from passively sensitized basophils, respectively. Identification of appropriate calibrators and predictive assays would allow for the future assessment of allergenicity of novel food proteins. Indeed, invertebrate TM is a known food allergen while only few studies relate vertebrate TM to clinical food allergy (eg in fish).^39^, ^40^, ^41^

First, we showed that the purified proteins from shrimp and chicken origin exhibit similar biomolecular properties. Allergen Pen m 1 was confirmed as a dimer made of two alpha‐helical chains. TM from chicken breast was found mostly as αα‐homodimer and TM from leg muscle as αβ‐heterodimer. Indeed, TM isoforms are known to originate from separate genes, vary across species and tissues.^42^ Shrimp Pen m 1 and the chicken homologues share protein identities between 55% and 59% (chicken TM β‐chain and α‐chain, respectively). Both TM from shrimp and chicken muscle were stable upon heating as defined by antibody‐based detection as well as monitoring by circular dichroism analysis. Thus, the thermal unfolding of the αβ‐heterodimeric proteins was found to be similar to that of the αα‐homodimers.^43^ Common food allergens are often more stable during thermal treatment than others.^19^, ^20^ However, both allergenic shrimp and non‐allergenic chicken TM showed no differences in thermal de‐/refolding characteristics.

Simulated digestion experiments revealed clear stability differences in shrimp and chicken TM. Assay conditions were chosen mild (pH 3; 1 U pepsin/µg extract protein) in order to follow the progressive protein digestion. Although in our study resistance to pepsinolysis separated the allergen from the non‐allergen, it is important to say that a straightforward correlation between protease stability and allergenicity is not always given, depending on the protein analysed and the assay conditions applied.^35^ Immunodetection of TM was performed using two different anti‐TM antibodies (recognizing possibly different TM epitopes). While shrimp Pen m 1 was detectable during the gastric phase, chicken homologues were degraded quickly during the first minutes of in vitro digest. This confirmed previous in silico and in vitro experiments showing high stability of allergenic versus low stability of non‐allergenic TM.^35^, ^44^ Indeed, the relative high stability of Pen m 1 towards protease degradation, in comparison with the invertebrate homologues is a plausible explanation for immunogenicity and allergenicity of the shrimp protein.^45^ In terms of exposure levels to shrimp and chicken TM, we estimated the protein content of TMs in total extracts by densitometric band quantification from SDS‐PAGE.^29^ Similar levels, ca. 10%‐12% TM of total extracted protein, were found for both shrimp and chicken TM (data not shown), indicating that exposure levels might be similar as well.

In order to assess the TMs’ allergenicity, the assays used needed to be performed with fully titrated protein amounts for the determination of semi‐quantitative relative potency, or as quantitative methods showing a quantitative response.

First, we assessed the quantitative IgE‐binding capacity of TM preparations, using IgE ELISA (Figure 2) in a shrimp‐allergic cohort of patients tolerating chicken meat. All patients had IgE to shrimp Pen m 1 (native, recombinant). Most patients (70%) had also specific IgE recognizing native chicken leg αβ‐TM. Five linear B cell‐epitopes have been reported for Pen m 1.^46^, ^47^ Comparing those epitope regions to homologue chicken TM peptides, we found a median protein identity of 60% for chicken αβ‐TM, respectively (Figure S2). Thus, IgE cross‐recognition can be explained by homology in epitope regions from both shrimp and chicken αβ‐TM. However, quantified IgE levels revealed a significant difference for the protein pair of shrimp and chicken αα‐TM, with clearly higher IgE levels for the food allergen Pen m 1. When comparing the median levels of specific IgE to natural shrimp and chicken TM using IgE ELISA, there was approximately one order of difference in relative potency. When comparing the levels of specific IgE to the recombinant shrimp and chicken TM preparations, the description of the relative potency of IgE binding to chicken TM failed because most of the sera tested negative, that is below the limit of detection (0.1 kU_A_/L) (Figure 2). Only 5/20 shrimp‐allergic patients showed IgE binding to natural or recombinant chicken αα‐TM. Because the differences in IgE binding to shrimp and chicken TM were too little or not quantifiable, the IgE ELISA appeared not qualified as a tool for the quantitative allergenicity assessment of the pair of TM proteins in terms of relative potency.

In this study, the in vivo IgE‐test, by titration of the skin reactivity towards diluted TM samples, in patients allergic to shrimp but non‐allergic to chicken, revealed that only the shrimp homologue induced positive skin responses, except for a single patient (weak positive skin test of 3 mm with chicken leg TM, at the highest concentration tested vs skin test positive with shrimp TM at 50 times lower concentration). Thus, it was not possible to describe, in a relative quantitative way, the difference in allergenic potency between shrimp and chicken TM. Moreover, skin testing as a functional assay for the allergenicity assessment of novel proteins may be limited for ethical reasons and limited availability of suitable allergic volunteers.

By contrast, the ex vivo cellular BAT using patients' sensitized basophils, as well as the in vitro RBL cell test, using an immortal rat basophil cell line transfected with the human FcεRI receptor and passively sensitized with patient sera, was positive with both shrimp and chicken TM. The titrated analysis of the proteins allowed for determination of a relative quantitative response. When comparing this relative potency, we further confirmed the low reactivity induced by chicken TM versus the shrimp allergen in both basophil tests. Importantly, the biological activity (basophil activation, release of mediators) was determined for shrimp Pen m 1 at up to 100 and respectively, 1000 times lower protein concentrations than with chicken homologues, using both human basophils of shrimp‐allergic subjects and RBL cells passively sensitized with IgE of shrimp‐allergic serum donors. Hence, the basophil activation test, which also is under discussion as a specific and sensitive diagnostic tool in the clinical management of food‐allergic patients,^48^, ^49^ can in principle be used for the allergenicity assessment of proteins if appropriate human basophil donors (‘responders’) have been identified. However, the need of fresh human material somewhat limits the use of BAT for allergenicity assessment of proteins. This turns the RBL cell test, based on an immortal humanized rat basophil cell line, in combination with patient sera for passive sensitization (preselected on Pen m 1‐specific IgE titres of >2 kU_A_/L), to a promising alternative assay, that can potentially be standardized, for the allergenicity assessment of novel proteins. As results for native and recombinant proteins (shrimp Pen m 1, chicken αα‐TM) were consistent in both functional assays (BAT, RBL), the recombinant counterparts were used as clinically validated standards of high and low allergenicity for the allergenicity assessment of a novel food protein, that is TM of yellow mealworm (*Tenebrio molitor*) as a model protein. While rPen m 1 and mealworm TM showed a very comparable and high allergenic activity on effector cells, r‐chicken αα‐TM had a low capacity to release mediators from basophils. Hence, the RBL cell assay using rPen m 1 and r‐chicken αα‐TM as calibrators confirmed mealworm TM, as one example of a newly introduced food protein with high allergenic potency that is comparable to that of shrimp tropomyosin. Indeed, yellow mealworm is a novel food that causes allergic reactions as a primary and as a cross‐reacting food allergen.^4^, ^26^, ^33^ As previously published, for the majority of shrimp‐allergic individuals, mealworm TM is a major allergen, and clinical reactivity to mealworm was in the same range as to shrimp, both with regard to severity of allergic reactions and eliciting doses.^26^ In this context, the outcome of reported clinical reactivity to mealworm^26^ and the semi‐quantitative in vitro allergenic potency of mealworm TM, as determined in this study in relation to clinically validated TM calibrants, are very much comparable.

Following available guidance on the allergenicity assessment of GM foods,^8^, ^28^, ^29^ the current weight‐of‐evidence approach is based on the safety evaluation including consideration of the gene's source, homology of the translated gene with known allergens, IgE‐binding tests with sera from allergic patients and proteolysis (pepsin) stability testing. With a sequence identity of greater than 35% to known crustacean allergens and positive IgE binding in sera from shrimp‐allergic subjects, chicken TM would have been classified as potentially allergenic protein according to this strategy and beyond, even the functional IgE testing (BAT, RBL) was positive. Essentially, only the comparison of the relative potency, by matching effector cell activation versus titrated protein concentrations, revealed the discrimination of the allergen from the non‐allergen. The titrated effector cell reactivity test based on homologues could be proposed to be included in current risk assessment procedures. For assay calibration, threshold definition for low and high allergenicity would be required. However, it needs to be considered that the performance of the mediator release is always related to the selected patients’ sera. Accordingly, appropriate ‘high performing’ sera would need to be identified, selected and further characterised with regard to repeatability of assay performance. Subsequently, appropriate and relevant cut‐off levels for positivity and range of calibrator amount can be defined, though this represents another level of assay validation.

To identify percentages of mediator release reflecting clinical reactivity or oral tolerance, future studies will be required including food challenges that allow to approach threshold reactivity in terms of ingested food protein doses—however, a true prediction of clinical reactivity, based on a cellular test, is expected to be unlikely. Further recommendations for using mediator release assays pertain to the novel food source that shall be tested also as a total extract, as this allows to target allergens beyond the calibrator molecules.

Finally, it is important to point out that the proposed calibration system is TM‐specific. With this TM model as a proof of concept, similar systems of homologous proteins might be elaborated based on other allergens/non‐allergens, such high and low allergenic nsLTP in plant foods. Taking together the data from our study using the cohort of shrimp‐allergic patients including the semi‐quantitative cellular test, mealworm (based on its TM) would have been ranked with an allergenic potential that is comparable high to that of shrimp while chicken meat (also, based on its TM) would have been assessed as a food with low allergenic potential for human consumption. The approach of protein pairs represents a perspective for the risk assessment of novel foods where homologous proteins to known strong or anaphylactic food allergens are present, and thus, the risk is related to sensitized/allergic individuals. De novo sensitization, possibly to proteins different from TM, would require further consideration in the allergenicity assessment guidance, a topic that goes beyond the present study.

In summary, this study shows that clinically validated tropomyosin from invertebrate and vertebrate origin, shrimp Pen m 1 and the chicken tropomyosin as a pair of homologues, share similar biomolecular characteristics but vary greatly in biological activity on effector cells of the immediate‐type allergic reaction. These protein homologues can be better standardized when used as recombinant proteins with definite and reproducible structural characteristics. The use of such candidate molecules may allow for standardization of functional assays to assess the allergenic potency of novel food proteins at the site of elicitation. In this context, the humanized RBL cell assay showed high potential in the allergenicity assessment of mealworm TM as a model novel food protein, when calibrated with the pair of allergenic and non‐allergic recombinant shrimp and chicken TM.

## CONFLICT OF INTEREST

All other authors have declared that they have no conflict of interest.

## Data Availability

The data that support the findings of this study are available on request from the corresponding author. The data are not publicly available due to privacy or ethical restrictions.
