# Supplementary material for: Homologous tropomyosins from vertebrate and invertebrate: Recombinant calibrator proteins in functional biological assays for tropomyosin allergenicity assessment of novel animal foods
Source: Clin Exp Allergy. 2019 Oct 11;50(1):105–16. doi: 10.1111/cea.13503 (PMC6973240; doi:10.1111/cea.13503)
Supplement: Supplementary file 1 [file CEA-50-105-s001.doc]

Title: **Homologous tropomyosins from vertebrate and invertebrate: calibrator proteins in new assays for food allergenicity assessment.**

Short running title: **Shrimp and chicken tropomyosins.**

*Online supporting information; table count: 7; figure count: 5.*

Julia Klueber^1,2^, MSc, Joana Costa^3^, PhD, Stefanie Randow^4^, Françoise Codreanu-Morel^5^, MD, Kitty Verhoeckx^6^, PhD, Carsten Bindslev-Jensen^2^, MD, PhD, Markus Ollert^1,2^, MD, DMSc, Karin Hoffmann-Sommergruber^7^, PhD, Martine Morisset^5*^, MD, PhD, Thomas Holzhauser^4#^, PhD, Annette Kuehn^1#^, PhD

^1^Department of Infection and Immunity, Luxembourg Institute of Health, Esch-sur-Alzette, Luxembourg; ^2^Department of Dermatology and Allergy Center, Odense Research Center for Anaphylaxis, University of Southern Denmark, Odense C, Denmark;

^3^REQUIMTE-LAQV/Faculdade de Farmácia da Universidade do Porto, Porto, Portugal; ^4^Division of Allergology, Paul-Ehrlich-Institut, Langen, Germany; ^5^National Unit of Immunology and Allergology, Centre Hospitalier de Luxembourg, Luxembourg;

^6^RAPID, TNO, Zeist, The Netherlands; ^7^Department of Pathophysiology and Allergy Research, Medical University of Vienna, Vienna, Austria.

*author’s present address, different to where the work was carried out: Unité d’Allergologie, CHU Angers, Angers, France

^#^Thomas Holzhauser and Annette Kuehn contributed equally to the study and thus, are joint senior authors.

**Corresponding author**: Annette Kuehn, PhD; Department of Infection and Immunity, Luxembourg Institute of Health, 29, rue Henri Koch, L-4354 Esch-sur-Alzette; phone: + 352 26970 335; fax: + 352 26970 390; E-mail: [annette.kuehn@lih.lu](mailto:annette.kuehn@lih.lu)

**TABLES**

**Table S1.** Demographic and clinical characteristics of controls.

| **No.** | **Gender/Age** | **Allergies** | **IgE-titers^1^ [kU/L]** |
| --- | --- | --- | --- |
|  |  |  | **Total** |
| 1 | F/28 | Grass pollen, tree nuts | 533 |
| 2 | F/42 | Grass pollen, molds, animal dander | 123 |

**Table S2. House dust mite allergy in shrimp-allergic patient cohort.**

| **No.** | **Gender/Age** | **HDM allergy** | **IgE-titers^1^ [kU_A_/L]** | |
| --- | --- | --- | --- | --- |
|  |  |  | **D. pter.** | **Der p 10** |
| 1 | M/17 | Y | 15 | 11 |
| 2 | M/9 | Y | 100 | 14 |
| 3 | M/17 | Y | 100 | 100 |
| 4 | M/16 | Y | 100 | 100 |
| 5 | M/6 | N | Neg | Neg. |
| 6 | M/10 | N | Neg | Neg |
| 7 | F/13 | Y | 12 | 4.5 |
| 8 | F/40 | Y | 0.9 | 1.8 |
| 9 | F/15 | Y | 46 | 27 |
| 10 | M/13 | Y | 23 | 1.2 |
| 11 | F/21 | Y | 61 | 21 |
| 12 | M/7 | Y | 2.5 | 1.9 |
| 13 | F/20 | Y | 4.6 | 9.2 |
| 14 | M/19 | Y | 100 | 80 |
| 15 | M/14 | Y | 55 | 0.5 |
| 16 | F/52 | N | 0.5 | 0.5 |
| 17 | M/41 | Y | 10 | 0.5 |
| 18 | M/15 | Y | 100 | 5.6 |
| 19 | F/16 | Y | 30 | 46 |
| 20 | F/52 | Y | 1 | 1.6 |
| Mean | 20.7 | - | 47.1 | 39.9 |
| Median | 16 | - | 38 | 24 |

HDM, house dust mite

^1^IgE-determination by ImmunoCAP: D. pter., *Dermatophagoides pteronyssinus* extract (d1); Der p 10, HDM tropomyosin (d205)

**Table S3.** Shrimp tropomyosin peptides matching with tropomyosin of *Penaeus monodon* (ADM34184.1) sequence after request in the NCBInr database.

| **Start-End** | **Observed** | **Mr, exp.** | **Mr, calc.** | **Miss** | **Sequence** |
| --- | --- | --- | --- | --- | --- |
| 5-11 | 864.349 | 863.341 | 863.479 | 2 | KKMQAMK |
| 7-20 | 1680.740 | 1679.733 | 1679.787 | 2 | MQAMKLEKDNAMDR |
| 21 - 34 | 1630.792 | 1629.784 | 1629.778 | 1 | ADTLEQQNKEANNR |
| 30 - 47 | 2124.977 | 2123.970 | 2124.027 | 2 | EANNRAEKSEEEVHNLQK |
| 35 - 48 | 1696.852 | 1695.844 | 1695.861 | 2 | AEKSEEEVHNLQKR |
| 38 - 48 | 1368.690 | 1367.683 | 1367.687 | 1 | SEEEVHNLQKR |
| 49 - 75 | 3169.451 | 3168.443 | 3168.641 | 2 | MQQLENDLDQVQESLLKANIQLVEKDK |
| 66 - 89 | 2553.238 | 2552.230 | 2552.363 | 2 | ANIQLVEKDKALSNAEGEVAALNR |
| 76 - 89 | 1414.721 | 1413.713 | 1413.728 | 0 | ALSNAEGEVAALNR |
| 76 - 90 | 1570.829 | 1569.821 | 1569.830 | 1 | ALSNAEGEVAALNRR |
| 91 - 104 | 1758.878 | 1757.871 | 1757.887 | 1 | IQLLEEDLERSEER |
| 91 - 111 | 2488.219 | 2487.211 | 2487.289 | 2 | IQLLEEDLERSEERLNTATTK |
| 112 - 127 | 1807.830 | 1806.822 | 1806.860 | 2 | LAEASQAADESERMRK Oxidation (M) |
| 112 - 126 | 1663.741 | 1662.733 | 1662.770 | 1 | LAEASQAADESERMR |
| 133 - 151 | 2234.024 | 2233.016 | 2233.072 | 2 | SLSDEERMDALENQLKEAR |
| 140 - 151 | 1417.666 | 1416.658 | 1416.710 | 1 | MDALENQLKEAR |
| 152 - 166 | 1811.885 | 1810.877 | 1810.892 | 2 | FLAEEADRKYDEVAR |
| 161 - 167 | 880.370 | 879.363 | 879.452 | 1 | YDEVARK |
| 167 - 181 | 1776.146 | 1775.138 | 1774.896 | 2 | KLAMVEADLERAEER Oxidation (M) |
| 178 - 188 | 1206.132 | 1205.124 | 1205.560 | 1 | AEERAETGESK |
| 182 - 204 | 2556.256 | 2555.249 | 2555.352 | 2 | AETGESKIVELEEELRVVGNNLK |
| 189 - 204 | 1854.007 | 1853.000 | 1853.033 | 1 | IVELEEELRVVGNNLK |
| 205 - 216 | 1389.678 | 1388.671 | 1388.697 | 1 | SLEVSEEKANQR |
| 222 - 232 | 1315.752 | 1314.744 | 1314.794 | 2 | EQIKTLTNKLK |
| 231 - 243 | 1461.790 | 1460.782 | 1460.781 | 2 | LKAAEARAEFAER |
| 233 - 243 | 1220.599 | 1219.591 | 1219.602 | 1 | AAEARAEFAER |
| 233 - 247 | 1662.818 | 1661.811 | 1661.856 | 2 | AAEARAEFAERSVQK |
| 238 - 250 | 1533.837 | 1532.830 | 1532.838 | 2 | AEFAERSVQKLQK |
| 238 - 247 | 1164.592 | 1163.584 | 1163.601 | 1 | AEFAERSVQK |
| 251 - 265 | 1844.894 | 1843.886 | 1843.924 | 2 | EVDRLEDELVNEKEK |
| 266 - 283 | 2095.944 | 2094.936 | 2094.971 | 1 | YKSITDELDQTFSELSGY |
|  |  |  |  |  |  |

Start-End, positions of the peptides in the protein sequence; Observed, experimental m/z value; Mr,exp., experimental m/z transformed to a relative molecular mass; Mr, calc., relative molecular mass calculated from the matched peptide sequence; Miss, number of missed cleavage site; Sequence, sequence of the peptide in one letter code; Oxidation (M), oxidation of the methionine amino-acid residue.

**Table S4.** Chicken leg tropomyosin (upper band) peptides matching with tropomyosin beta chain of *Gallus gallus* (P19352.1) sequence after request in the NCBInr database.

| **Start-End** | **Observed** | **Mr, exp.** | **Mr, calc.** | **Miss** | **Sequence** |
| --- | --- | --- | --- | --- | --- |
| 8-21 | 1704.916 | 1703.908 | 1703.877 | 2 | MQMLKLDKENAIDR |
| 30 - 37 | 977.380 | 976.372 | 976.483 | 2 | KQAEDRCK |
| 31 - 48 | 2160.074 | 2159.066 | 2159.035 | 2 | QAEDRCKQLEEEQQGLQK |
| 36 - 48 | 1560.802 | 1559.794 | 1559.769 | 1 | CKQLEEEQQGLQK |
| 36 - 49 | 1688.894 | 1687.886 | 1687.864 | 2 | CKQLEEEQQGLQKK |
| 38 - 49 | 1457.787 | 1456.779 | 1456.759 | 1 | QLEEEQQGLQKK |
| 77 - 90 | 1474.803 | 1473.796 | 1473.750 | 1 | KATDAEAEVASLNR |
| 77 - 91 | 1630.889 | 1629.881 | 1629.851 | 2 | KATDAEAEVASLNRR |
| 78 - 91 | 1502.797 | 1501.789 | 1501.756 | 1 | ATDAEAEVASLNRR |
| 91 - 105 | 1884.034 | 1883.026 | 1882.993 | 2 | RIQLVEEELDRAQER |
| 92 - 105 | 1727.933 | 1726.925 | 1726.892 | 1 | IQLVEEELDRAQER |
| 102 - 118 | 1928.515 | 1927.507 | 1927.045 | 2 | AQERLATALQKLEEAEK |
| 106 - 125 | 2202.159 | 2201.151 | 2201.125 | 2 | LATALQKLEEAEKAADESER |
| 119 - 133 | 1704.916 | 1703.908 | 1703.833 | 2 | AADESERGMKVIENR |
| 126 - 133 | 946.515 | 945.507 | 945.514 | 1 | GMKVIENR |
| 129 - 140 | 1477.842 | 1476.834 | 1476.732 | 2 | VIENRAMKDEEK Oxidation (M) |
| 134 - 140 | 866.290 | 865.282 | 865.392 | 1 | AMKDEEK Oxidation (M) |
| 134 - 149 | 1980.980 | 1979.973 | 1979.944 | 2 | AMKDEEKMELQEMQLK |
| 141 - 152 | 1477.842 | 1476.834 | 1476.739 | 1 | MELQEMQLKEAK |
| 153 - 167 | 1815.939 | 1814.931 | 1814.898 | 2 | HIAEEADRKYEEVAR |
| 162 - 168 | 894.234 | 893.226 | 893.468 | 1 | YEEVARK |
| 168 - 182 | 1786.003 | 1784.995 | 1784.971 | 2 | KLVVLEGELERSEER |
| 168 - 178 | 1284.792 | 1283.784 | 1283.752 | 1 | KLVVLEGELER |
| 169 - 182 | 1657.903 | 1656.895 | 1656.876 | 1 | LVVLEGELERSEER |
| 206 - 220 | 1712.448 | 1711.440 | 1711.834 | 2 | SLEAQADKYSTKEDK |
| 214 - 220 | 870.527 | 869.519 | 869.420 | 1 | YSTKEDK |
| 232 - 244 | 1549.840 | 1548.832 | 1548.797 | 2 | LKEAETRAEFAER |
| 234 - 244 | 1308.669 | 1307.662 | 1307.618 | 1 | EAETRAEFAER |
| 239 - 251 | 1477.842 | 1476.834 | 1476.801 | 2 | AEFAERSVAKLEK |
| 252 - 266 | 1797.889 | 1796.881 | 1796.858 | 1 | TIDDLEDEVYAQKMK |
| 252 - 268 | 2089.057 | 2088.049 | 2088.016 | 2 | TIDDLEDEVYAQKMKYK |
| 265 - 284 | 2268.160 | 2267.152 | 2267.143 | 2 | MKYKAISEELDNALNDITSL |
| 267 - 284 | 2009.043 | 2008.035 | 2008.007 | 1 | YKAISEELDNALNDITSL |
|  |  |  |  |  |  |

Start-End, positions of the peptides in the protein sequence; Observed, experimental m/z value; Mr,exp., experimental m/z transformed to a relative molecular mass; Mr, calc., relative molecular mass calculated from the matched peptide sequence; Miss, number of missed cleavage site; Sequence, sequence of the peptide in one letter code; Oxidation (M), oxidation of the methionine amino-acid residue.

**Table S5.** Chicken leg tropomyosin (lower band) peptides matching with Tropomyosin alpha-1 chain of *Gallus gallus* (P04268.2) sequence after request in the NCBInr database.

| **Start-End** | **Observed** | **Mr, exp.** | **Mr, calc.** | **Miss** | **Sequence** |
| --- | --- | --- | --- | --- | --- |
| 8-21 | 1704.921 | 1703.914 | 1703.877 | 2 | MQMLKLDKENALDR |
| 22 - 35 | 1545.784 | 1544.776 | 1544.750 | 2 | AEQAEADKKAAEER |
| 36 - 48 | 1500.841 | 1499.833 | 1499.827 | 1 | SKQLEDELVALQK |
| 36 - 49 | 1628.949 | 1627.941 | 1627.922 | 2 | SKQLEDELVALQKK |
| 38 - 49 | 1413.808 | 1412.800 | 1412.795 | 1 | QLEDELVALQKK |
| 52 - 70 | 2185.190 | 2184.182 | 2184.014 | 2 | GTEDELDKYSESLKDAQEK |
| 77 - 91 | 1646.884 | 1645.876 | 1645.846 | 2 | KATDAESEVASLNRR |
| 78 - 91 | 1518.782 | 1517.774 | 1517.751 | 1 | ATDAESEVASLNRR |
| 91 - 105 | 1884.028 | 1883.020 | 1882.993 | 2 | RIQLVEEELDRAQER |
| 92 - 105 | 1727.919 | 1726.912 | 1726.892 | 1 | IQLVEEELDRAQER |
| 102 - 118 | 1928.535 | 1927.527 | 1927.045 | 2 | AQERLATALQKLEEAEK |
| 119 - 133 | 1704.921 | 1703.914 | 1703.833 | 2 | AADESERGMKVIENR |
| 126 - 133 | 946.509 | 945.501 | 945.514 | 1 | GMKVIENR |
| 134 - 149 | 1960.034 | 1959.027 | 1959.006 | 2 | AQKDEEKMEIQEIQLK |
| 153 - 167 | 1815.927 | 1814.919 | 1814.898 | 2 | HIAEEADRKYEEVAR |
| 168 - 182 | 1770.003 | 1768.995 | 1768.976 | 2 | KLVIIEGDLERAEER |
| 168 - 178 | 1284.783 | 1283.775 | 1283.752 | 1 | KLVIIEGDLER |
| 206 - 213 | 875.486 | 874.478 | 874.447 | 0 | SLEAQAEK |
| 232 - 244 | 1549.827 | 1548.819 | 1548.797 | 2 | LKEAETRAEFAER |
| 234 - 244 | 1308.653 | 1307.646 | 1307.618 | 1 | EAETRAEFAER |
| 239 - 251 | 1507.854 | 1506.846 | 1506.811 | 2 | AEFAERSVTKLEK |
| 245 - 264 | 2324.208 | 2323.200 | 2323.187 | 2 | SVTKLEKSIDDLEDELYAQK |
| 252 - 268 | 2071.076 | 2070.068 | 2070.059 | 2 | SIDDLEDELYAQKLKYK |
| 252 - 266 | 1779.922 | 1778.914 | 1778.901 | 1 | SIDDLEDELYAQKLK |
| 267 - 284 | 2049.993 | 2048.985 | 2048.980 | 1 | YKAISEELDHALNDMTSI |
| 269 - 284 | 1758.842 | 1757.835 | 1757.821 | 0 | AISEELDHALNDMTSI |
|  |  |  |  |  |  |

Start-End, positions of the peptides in the protein sequence; Observed, experimental m/z value; Mr,exp., experimental m/z transformed to a relative molecular mass; Mr, calc., relative molecular mass calculated from the matched peptide sequence; Miss, number of missed cleavage site; Sequence, sequence of the peptide in one letter code.

**Table S6.** IgE-quantification by ELISA using extracts and purified molecules from shrimp, chicken and mealworm.

| **No.** | **Gender/Age** | **Shrimp: IgE-titers [kU_A_/L]** | | | **Chicken: IgE-titers [kU_A_/L]** | | | | **Mealworm:**  **IgE-titers [kU_A_/L]** |
| --- | --- | --- | --- | --- | --- | --- | --- | --- | --- |
|  |  | **Extract** | **Pen m 1 (n)** | **Pen m 1 (r)** | **Extract** | **αβ-TM (n)** | **αα-TM (n)** | **αα-TM (r)** | **TM (n)** |
| 1 | M/17 | 70 | 55 | 68 | 0.1 | 12 | 4.5 | 4.0 | 31 |
| 2 | M/9 | 13 | 10.4 | 8.5 | 0.1 | 1.8 | 0.1 | 0.1 | 2.5 |
| 3 | M/17 | 4 | 4 | 3.6 | 0.1 | 0.6 | 0.1 | 0.1 | 2.1 |
| 4 | M/16 | 9.4 | 7.5 | 5.9 | 0.4 | 5.4 | 1.1 | 1.9 | 4.1 |
| 5 | M/6 | 34 | 30 | 28 | 0.1 | 8.5 | 5.4 | 3.5 | 26 |
| 6 | M/10 | 2 | 4.3 | 2.9 | 0.1 | 0.5 | 0.1 | 0.1 | 0.5 |
| 7 | F/13 | 4.8 | 6.1 | 1.3 | 0.1 | 0.3 | 0.1 | 0.1 | 2.4 |
| 8 | F/40 | 1.1 | 2.3 | 0.9 | 0.1 | 0.1 | 0.1 | 0.1 | 1.4 |
| 9 | F/15 | 35 | 45 | 45 | 1.8 | 2.8 | 0.1 | 0.1 | 40 |
| 10 | M/13 | 0.6 | 0.7 | 0.3 | 0.1 | 0.1 | 0.1 | 0.1 | 0.9 |
| 11 | F/21 | 100 | 100 | 100 | 0.1 | 20.4 | 8.9 | 10.7 | 100 |
| 12 | M/7 | 1.5 | 3.9 | 1.5 | 0.1 | 0.3 | 0.1 | 0.1 | 3.1 |
| 13 | F/20 | 31 | 32 | 30 | 0.1 | 3.8 | 0.1 | 0.1 | 29 |
| 14 | M/19 | 100 | 100 | 100 | 0.1 | 7.2 | 0.1 | 0.1 | 100 |
| 15 | M/14 | 0.2 | 0.2 | 0.2 | 0.1 | 0.1 | 0.1 | 0.1 | 0.1 |
| 16 | F/52 | 0.7 | 0.2 | 0.2 | 0.1 | 0.1 | 0.1 | 0.1 | 0.3 |
| 17 | M/41 | 0.2 | 0.2 | 0.2 | 0.1 | 0.1 | 0.1 | 0.1 | 0.1 |
| 18 | M/15 | 0.4 | 1.4 | 0.7 | 0.1 | 0.1 | 0.1 | 0.1 | 1.0 |
| 19 | F/16 | 21.5 | 27.4 | 18.0 | 0.3 | 5.5 | 1.8 | 2.0 | 32.1 |
| 20 | F/52 | 7.7 | 3.5 | 2.2 | 3.1 | 0.3 | 0.1 | 0.1 | 3.4 |
| Mean | 20.7 | 21.9 | 21.7 | 20.9 | 0.4 | 3.5 | 1.2 | 1.2 | 19 |
| Median | 16 | 6.3 | 5.2 | 3.3 | 0.1 | 0.6 | 0.1 | 0.1 | 2.8 |

N, native protein; Pen m 1, shrimp tropomyosin; r, recombinant protein; TM, tropomyosin.

**Table S7.** Skin testing using native tropomyosins purified from shrimp and chicken.

| **No.** | **Gender/Age** | **TM** | **Skin testing, wheal [mm] at protein conc. [µg/ml]** | | | |
| --- | --- | --- | --- | --- | --- | --- |
|  |  |  | **0.1** | **1** | **10** | **50** |
| 1 | M/17 | Shrimp  Chicken | Neg  Neg | 5  Neg | -  Neg | -  3 |
| 2 | M/9 | Shrimp  Chicken | Neg  Neg | Neg  Neg | Neg  Neg | 7  Neg |
| 3 | M/17 | Shrimp  Chicken | Neg  Neg | Neg  Neg | Neg  Neg | 5  Neg |
| 4 | M/16 | Shrimp  Chicken | Neg  Neg | 4  Neg | 5  Neg | -  Neg |
| 6 | M/10 | Shrimp  Chicken | Neg  Neg | Neg  Neg | 4  Neg | -  Neg |
| 7 | F/13 | Shrimp  Chicken | Neg  Neg | Neg  Neg | 4  Neg | 12  Neg |

-, not done (skin testing discontinued when result positive at preceding protein conc.); conc., concentration; Neg, negative; TM, tropomyosin.

**Figure 1-SI. A.** Non-/heated shrimp and chicken extracts revealed similar protein profiles, tropomyosins turned to be heat-stable. **B.** Commercial antibodies detected shrimp and chicken tropomyosins in both non-/heated extracts. shrimp; leg, chicken leg; breast, chicken breast; arrow, tropomyosin signal; E, extract; ΔT, heated sample.


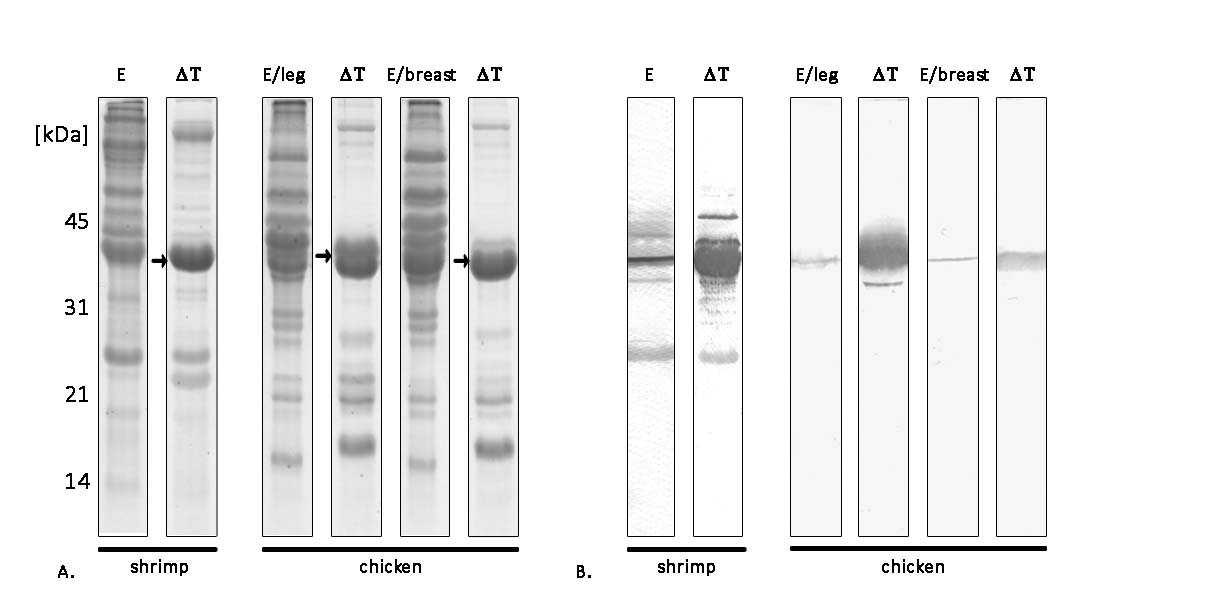


**Figure 2-SI.** Protein alignment analysis (BioEdit sequence alignment editor) showed high overall protein identity between shrimp Pen m 1 and chicken TMs (59 % for α-TM and 55 % for β-TM, respectively), including five Pen m 1 epitopes regions (E1, 33 and 27 %; E2, 71 %; E3, 60 %; E4, 67 and 60 %; E5, 46 and 43 % identity for α-TM and β-TM, respectively).

E2

E3

E4

E5

E1

**Figure 3-SI.** Circular dichroism analysis detected alpha-helical structures and thermal stability for shrimp and chicken tropomyosins. For all 5 proteins (nPen m 1, rPen m 1, n-chicken αα, n-chicken αβ, rec-chicken αα) CD-curves are shown grouped per assay condition (5 curves per condition within range of vertical bars), at room temperature (RT), upon heating (95°C) and after cooling back (RT back). Median values, range of data variation.

**Figure 4-SI. A.** The antibody-based signal for shrimp tropomyosin natural Pen m 1 was detectable during the entire gastric phase. **B.** The antibody-based signal for natural chicken tropomyosin disappeared quickly after five minutes of gastric digest. E, extract (all samples with digested extract protein); T0-T120, gastric digest at 0 to 120 min; arrow, tropomyosin signal.


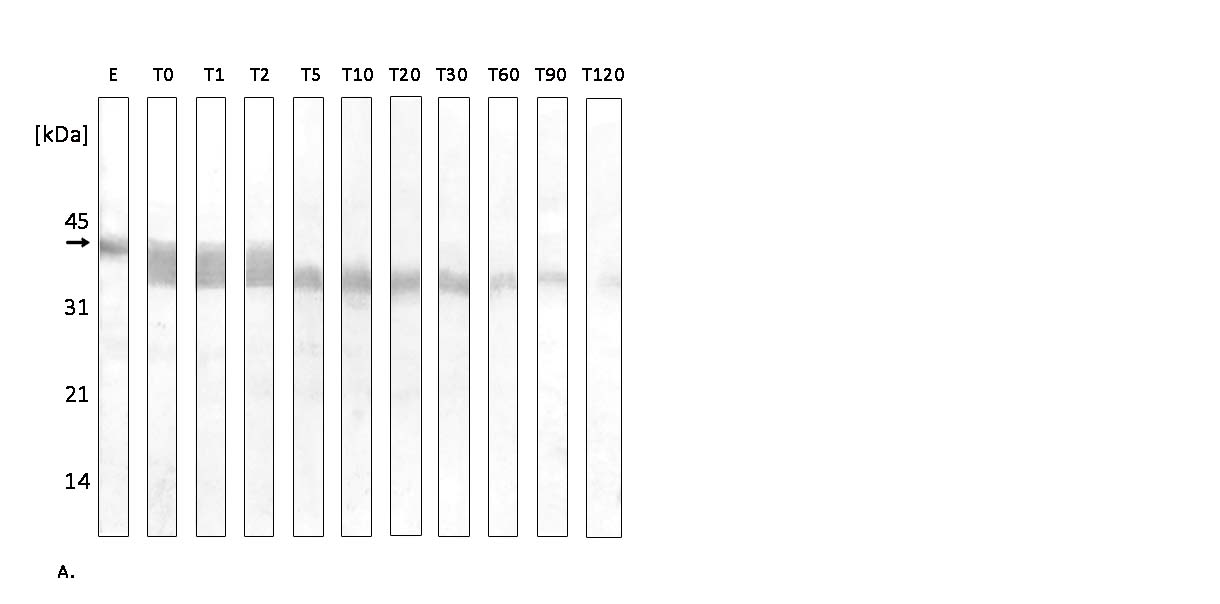


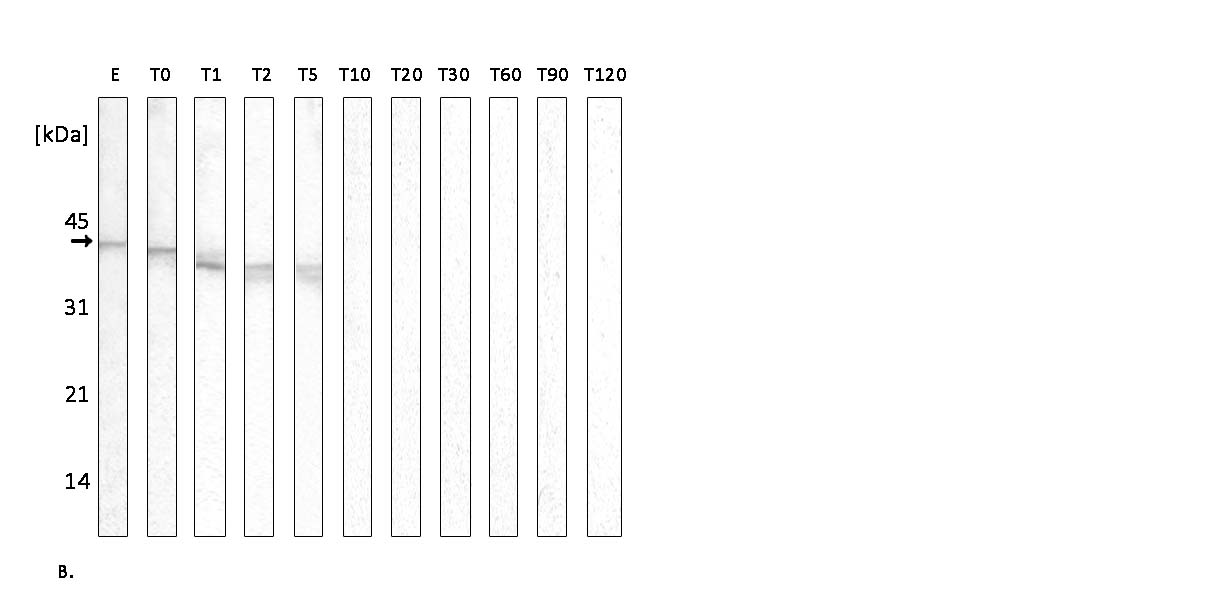


**Figure 5-SI. IgE Immunoblot analysis of shrimp total protein extract.** Shrimp allergens were detected by patients’ IgE-antibodies (except patient no. 3, serum depleted). Most sera (18/20) were positive for IgE-binding to ca. 35 kDa-tropomyosin, several patient sera (1, 2, 5, 11, 13, 14, 19, 20) recognized additional proteins. Arrow, tropomyosin recognized by commercial antibody.

**
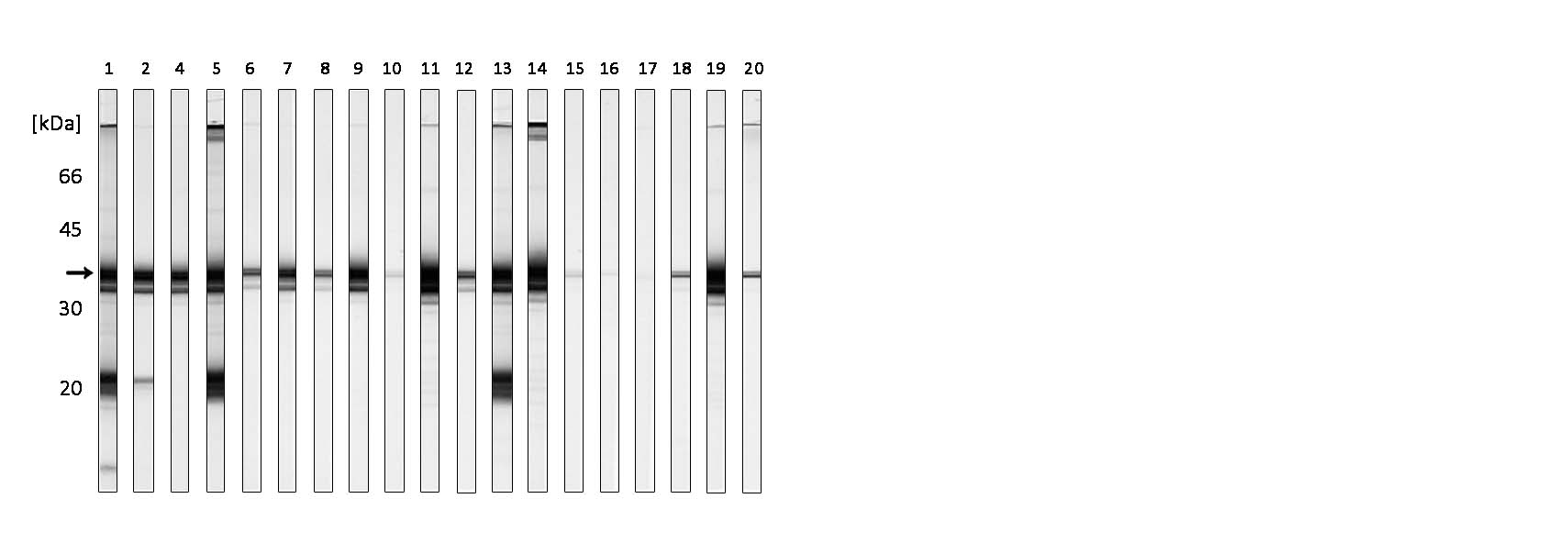
**
